# Interdisciplinary Hospice Clinician Presence During Patient Medical Aid in Dying Self-Administration

**DOI:** 10.1093/geroni/igaf122.869

**Published:** 2025-12-31

**Authors:** Todd Becker, Denae Gerasta, Grant Yoder, Daniel Matlock, Elissa Kozlov, Stacy Fischer, Karla Washington

**Affiliations:** WashU Medicine, St. Louis, Missouri, United States; University of Colorado Anschutz, Aurora, Colorado, United States; University of Colorado School of Medicine, Aurora, Colorado, United States; University of Colorado Anschutz Medical Campus, Aurora, Colorado, United States; Rutgers School of Public Health, Piscataway, New Jersey, United States; UCHealth University of Colorado Hospital (UCH), Aurora, Colorado, United States; Washington University in St. Louis, St. Louis, Missouri, United States

## Abstract

Many older adults using medical aid in dying (MAID) have reported a preference to have clinicians present while they self-administer medication to hasten death. However, limited data exist to characterize hospice clinician involvement during this time. This study aimed to examine correlates of hospice clinician presence during patient MAID self-administration. This secondary analysis used cross-sectional data from our earlier survey of hospice physicians, nurses, social workers, and chaplains. This convenience sample was recruited through national hospice and palliative care professional membership associations. We restricted the analytic sample to participants working for hospices servicing states where MAID is legal and permitting at least partial MAID participation (N = 100). We assessed personal, professional, organizational, and MAID-specific factors as correlates of ever having been present during patient MAID self-administration via a binary logistic regression model adjusted for small-sample bias. Results indicated that being a chaplain (vs. physician: OR = 5.29, p = .017) and working for a hospice permitting full MAID participation (vs. partial: OR = 4.84, p = .004) were each significantly associated with greater odds of being present during MAID self-administration. Findings represent the first exploration of factors associated with hospice clinician presence during patient MAID self-administration. Differences between chaplains and physicians may reflect discipline-specific skill sets corresponding to involvement at distinct points across the delivery of care. Conversely, hospices permitting full participation may be more amenable—or even encourage—clinician presence when requested. Future studies should examine how clinician roles and hospice policy shape patient perceptions of care receipt.

